# Multidecadal High Mortality Disease Events in Australian Domestic Geese Associated with a Novel Alphaherpesvirus, Designated Anatid Alphaherpevirus 2

**DOI:** 10.1155/2024/3255966

**Published:** 2024-06-07

**Authors:** Mirrim Kelly-Bosma, Selina Ossedryver, Rachel Olive Bowater, Jeff Butler, Tristan Reid, Willy W. Suen, Darren Underwood, Shaylie Latimore, Leonard Izzard, Ancy Joseph, Patrick Mileto, Vittoria Stevens, Jianning Wang, Tessa Mackie, Alexandr Mastakov, Robert Doneley, Rachel Allavena, Matthew J. Neave

**Affiliations:** ^1^ School of Veterinary Science The University of Queensland Gatton QLD 4343Australia; ^2^ IDEXX Laboratories Pty Ltd. 10 Brandl Street, Eight Mile PlainsQLD4113Australia; ^3^ Department of Agriculture and Fisheries Biosecurity Sciences Laboratory Coopers Plains QLD 4108Australia; ^4^ College of Public Health Medical and Veterinary Sciences James Cook University Townsville QLD 4814Australia; ^5^ Australian Centre for Disease Preparedness CSIRO East Geelong VIC 3220Australia

## Abstract

Herpesviruses are ubiquitous viruses which infect a wide range of hosts. Novel herpesviruses are being increasingly detected in free-ranging bird populations and there are growing concerns for cross-species infection and spillover events. Herein, multiple sporadic outbreaks of mortality caused by a herpesvirus are described in domestic geese in Queensland, Australia. Goose herpesvirus was initially detected in 1989 in south-east Queensland, and this article details four further recent outbreaks and reports novel genome sequencing and phylogeny of the preliminarily designated anatid alphaherpesvirus 2 (AnHV-2). Affected flocks were housed outdoors and comingled with other domesticated and wild anseriforms and other birds which were unaffected by disease. Affected geese displayed anorexia, lethargy, weakness, vomiting, and diarrhoea prior to death within 12–24 hr of the onset of clinical signs. Post-mortem examinations showed variable hepatic necrosis, splenic necrosis, fibrinonecrotising enteritis, lymphoid necrosis, necrotising thymitis, necrotising adrenalitis, and vasculitis. Intranuclear inclusion bodies were observed in hepatocytes, biliary epithelium, small intestinal mucosal epithelium, thymus, endothelial cells, ovarian stromal cells, adrenal cortical cells, and neuronal cell bodies in peripheral nerve ganglia. Transmission electron microscopy visualised herpesviral particles in virus culture supernatant, and within the nuclei of hepatocytes, biliary epithelium, and endothelial cells in case tissues. The genome sequence of this herpesvirus, designated anatid alphaherpesvirus 2 (AnHV-2), is described. While investigating goose mortalities, archived isolate from a swan with suspected herpesvirus infection was tested and genome sequencing identified a further novel herpesvirus, proposed anatid alphaherpesvirus 3 (AnHV-3). The AnHV-2 and AnHV-3 genomes were more similar to each other, with a nucleotide identity of 76.1%, than to reference genome sequences. Phylogenetically, the new genomes formed a distinct clade within the alphaherpesvirus genus *Mardivirus*. We sequenced four AnHV-2 genomes from different cases and these did not display consistent divergence over time or distance. Expanded surveillance and outbreak testing are recommended, facilitated by the development of a specific real-time PCR for the rapid detection of AnHV-2.

## 1. Introduction

*Herpesviridae* is a large family of ubiquitous viruses characterised by a 110–130 nm icosahedral capsid consisting of 162 capsomers, and a lipid bilayer envelope embedded with glycoproteins. Including the envelope, the virions can measure up to 220 nm in diameter [1]. This ultrastructure is distinctive on negative contrast and thin section electron microscopy. Members of this family are also known as herpesviruses. There are currently 115 recognised species of herpesviruses, which are categorised into three major subfamilies: *Alpha*-, *Beta*-, and *Gamma-herpesvirinae* [2].

Herpesviruses can infect a range of animals, including humans, birds, fish, and reptiles, with several well-described in avian hosts. All sequenced avian herpesviruses to date belong to the *Alphaherpesvirinae* subfamily, within the genera *Iltovirus* and *Mardivirus* [1]. *Iltovirus* contains gallid alphaherpesvirus 1, the cause of infectious laryngotracheitis (ILT) in poultry, and Marek's disease is caused by gallid alphaherpesvirus 2, a member of Mardivirus. In addition to the well-known avian herpesviruses, there is a recent increase in the detection of novel herpesvirus species in wild bird populations, as well as detection of herpesviruses in a wider range of avian hosts [3, 4, 5].

Whilst herpesviruses are generally considered host-specific, there are frequent instances of cross-species infection causing disease. Alphaherpesviruses in particular appear prone to crossing species barriers [6]. Some of these are accidental or “dead-end” hosts in which the virus cannot be further transmitted, such as suid alphaherpesvirus 1 causing Aujeszky's disease (pseudorabies) in a range of species other than swine [6, 7, 8]. Infection of an accidental host is usually fatal. Other herpesviruses appear to have multiple distantly related definitive hosts, such as equine alphaherpesvirus 9 [9]. Specifically in birds, there are multiple reports of pigeon herpesvirus (columbid alphaherpesvirus 1; CoHV-1) infection in birds of prey species [10, 11, 12].

There are two documented herpesviruses that cause disease in domestic geese (*Anser anser domesticus*). The most well-known of these is anatid alphaherpesvirus 1 (AnHV-1), the cause of duck plague (duck viral enteritis; DVE). AnHV-1 causes DVE in susceptible Anseriformes, with natural infection reported in wild and domestic species of ducks, geese, and swans of all ages, where it can cause up to 100% mortality. The virus targets gastrointestinal mucosal epithelial cells, lymphoid tissues, and endothelial cells, resulting in necrotising gastroenteritis, lymphoid necrosis, and systemic vasculitis with haemorrhages in various tissues [13, 14]. Migratory waterfowl are considered to play a crucial role in disease transmission within and between continents, with the disease said to have a global distribution [13]. Infection with AnHV-1 is listed as nationally notifiable in both Australia and New Zealand, where no cases of DVE have been reported previously.

The other herpesvirus reported to affect geese is simply referred to as “goose herpesvirus” in a single documented outbreak occurring in January 1989, in Queensland, Australia [15]. Affected geese died over a 24-day period, exhibiting weakness, prostration, and rapid death, with a mortality rate of 97%. Pathology findings included necrotising enteritis and hepatic necrosis with intranuclear inclusion bodies, and virions consistent with herpesvirus were observed on electron microscopy [15]. Ten years later, this goose herpesvirus isolate (GHV 552/89) was confirmed to be genetically distinct from DVE [16]. No further reports of similar disease incidents in geese attributable to goose herpesvirus have been published since the first outbreak.

This paper describes a series of four more recent outbreaks of highly fatal disease in domestic geese in Queensland, Australia, and provides results of genome sequencing and phylogenetic analysis which confirm the aetiological agent to be the same goose herpesvirus isolated in the 1989 outbreak. We propose to name the virus anatid alphaherpesvirus 2 (AnHV-2) in line with the International Committee on Taxonomy of Viruses (ICTV) herpesvirus guidelines (ICTV, 2021). We also describe a novel herpesvirus isolated from a black swan during the course of the investigation, preliminarily designated anatid alphaherpesvirus 3 (AnHV-3).

## 2. Materials and Methods

### 2.1. Case Material

Historical case (Lowood, 1989): Archived viral isolate (GHV 552/89) was stored frozen at the Australian Centre for Disease Preparedness (ACDP) and revived in 2021 for use in the present study.

Case 1 (Townsville, 2013): One goose was submitted to the College of Public Health, Medical and Veterinary Sciences, James Cook University (JCU), Veterinary Diagnostic Pathology Services for necropsy and diagnostic investigation. Following retrospective identification of this case in 2022, a stored paraffin-embedded block containing a piece each of liver, spleen, and intestine, was provided to ACDP for this study.

Case 2 (Maryborough, 2017): Two geese were necropsied by a local private veterinarian and a range of fresh and fixed tissues and tissue swabs were submitted to the Biosecurity Sciences Laboratory (BSL), Brisbane, for diagnostic investigation. An unidentified virus was isolated from the livers of both geese and forwarded to the ACDP for further investigation. Two herpesviral isolates, which had been stored frozen at the ACDP, were revived in 2022 for this study.

Case 3 (Townsville, 2020): Two geese were submitted for necropsy and diagnostic investigation to JCU, Veterinary Diagnostic Pathology Services. Frozen spleen and liver from one goose were submitted to BSL for investigation of suspected herpes viral infection and exclusion of duck viral enteritis (DVE). An unidentified virus was isolated and held frozen at BSL. In 2022, subsamples of fresh spleen, liver, and the unidentified viral isolate from both spleen and liver were forwarded from BSL to the ACDP for further molecular work in this study.

Case 4 (Lockyer Valley, 2021): Six geese were submitted to the School of Veterinary Science, The University of Queensland, Gatton, for necropsy and diagnostic investigation. Unpreserved liver and spleen from one bird were forwarded to the BSL for further work-up and exclusion of reportable diseases. Subsamples of unpreserved liver and spleen were sent from the BSL to the ACDP for exclusion of DVE and duck virus hepatitis (DVH). Virus was isolated concurrently at the BSL and the ACDP from the unpreserved spleen. The ACDP isolate underwent additional genomic analysis as reported here. Formalin-fixed liver was subsequently provided to ACDP in 2022 for use in this present study.

Swan case (Cairns, 1991): Two black swans were submitted to IDEXX (formerly VPS), Brisbane, for necropsy and histopathology. Frozen liver and kidney from one bird were forwarded to the BSL for virology. An unidentified herpesvirus isolated from primary chicken kidney cell culture was subsequently forwarded to the ACDP. Virus archived at ACDP was revived in 2022 for this study.

Detailed clinicopathologic and diagnostic test findings are provided in Tables S1 and S2.

### 2.2. Pathology

Routine post-mortem examination was performed on submitted whole geese in Cases 1, 3, and 4 (Table S1). A mix of fresh chilled and frozen geese were necropsied. A range of tissues were fixed in 10% neutral buffered formalin for histopathology. Chilled tissue and cloacal and tracheal swabs were taken for molecular diagnostics and virus isolation. Tissues for histopathology were routinely processed and slides were stained with haematoxylin and eosin using a standard method [17].

### 2.3. Molecular Diagnostics

Potential viral pathogens were investigated using standard molecular techniques (Table S2). At BSL, total nucleic acid was extracted from a cloacal and a tracheal swab from Case 4 using the MagMAX-96 Viral RNA Isolation Kit (Thermo Fisher Scientific) with a MagMAX Express-96 Magnetic Particle Processor (Thermo Fisher Scientific). Swabs were vortexed in 3 mL of viral transport medium (VTM—SPGA) and 100 *µ*L of sample homogenate added to the lysis buffer mix for robotic extraction as per the manufacturer's instructions. Real-time PCR analysis was carried out for exclusion of Avian influenza virus type A (AIV) and Avian paramyxovirus 1 (APMV-1) also known as Newcastle disease virus (NDV). For AIV exclusion, the TaqMan real-time PCR developed by Spackman et al. [18] and modified by Heine et al. [19] with the inclusion of an internal control was run at BSL on the Rotor-Gene platform (QIAGEN) [18, 19]. Duplicate 25 *µ*L reactions containing SuperScript III One-step PCR Platinum Taq Master Mix were amplified with cycling parameters consisting of a reverse transcription at 45°C for 10 min, Taq activation at 95°C for 2 min and 45 cycles of 95°C for 15 s and 60°C for 45 s. APMV-1 exclusion utilised a real-time PCR targeting the F-gene based on Australian isolates along with a multiplex PCR targeting the L-gene of Class I NDVs [20] and M-gene of Class II NDVs [21]. A 25 *µ*L reaction volume was used for both NDV assays and the cycling parameters for the L-gene and M-gene multiplex assay consisted of 50°C for 15 min, 95°C for 2 min and 45 cycles of 95°C for 10 s and 60°C for 45 s. A three-step cycling was used for the F-gene assay consisting of 50°C for 15 min, 95°C for 2 min and 45 cycles of 95°C for 10 s, 52°C for 30 s, and 72°C for 15 s.

A range of potential viral pathogens were variably excluded at the time of each submission to ACDP by real-time PCR on a range of fresh tissue and swab samples. Assays used for this purpose included Influenza A TaqMan PCR [18, 19], Newcastle Disease virus ML multiplex [21] and F gene [22] TaqMan PCRs, Duck enteritis virus TaqMan PCR [23], Duck hepatitis virus 1 TaqMan PCR (Wang personal communication), Pigeon rotavirus TaqMan PCR (Wang personal communication), and Duck astrovirus 1 and 2 TaqMan PCR (Wang personal communication).

### 2.4. Pan-Herpesvirus Conventional PCR

DNA was extracted from 50 *μ*L of supernatant from a 10% tissue homogenate using the MagMAX-96 Viral RNA Isolation Kit and processed with the MagMAX Express 24 (ThermoFisher). Extracted DNA was used for PCR testing immediately or stored at −80°C for further use.

A pan-herpesvirus PCR was used for initial detection and identification of the viruses [24]. The PCR was performed using HotStarTaq Plus Master Mix (Qiagen) according to the manufacturer's instructions. Each PCR reaction consisted of 12.5 *μ*L of 2x Taq polymerase buffer, 2.5 *μ*L of CoralLoad concentrate (10x), 0.5 *μ*L (20 *μ*M) of each forward and reverse primer and 5 *μ*L of DNA. For nested PCR, 2 *μ*L of first-round PCR product was used. Thermocycling conditions for both PCRs were 95°C for 5 min, then 30 cycles of 95°C for 30 s, 46°C for 60 s, and 72°C for 60 s. PCR products were purified using the QIAQuick gel purification system (Qiagen) and sequenced with the BigDye terminator v3.1 kit on a 3500 Genetic Analyser (Applied Biosystems). Sequences were analysed using Geneious Prime v.2020.2.4.

### 2.5. Quantitative PCR (qPCR) for AnHV-2

For rapid and specific detection of AnHV-2, a real-time quantitative TaqMan PCR assay was developed (Table 1). The primers and probe were designed based on the polymerase gene, obtained by initial conventional PCR and subsequent NGS analysis, using Primer Express 3 (Applied Biosystems). Sequences of the primers and probe were forward primer (GHV-F), 5′-GGC GAA CGG CCT GTT G-3′; reverse primer (GHV-R), 5′-TGA ATA TAG TCG CGT ACC GAC AA-3′; probe (GHV-P), 5′-FAM—ACC GTC ACC/ZEN/ACG ATA GGT CGC AAC A-3′-IABkFQ (5′ nuclease double-quenched probe).

The PCR was performed in 15 *μ*L reaction volumes containing 5 *μ*L of DNA, 7.5 *μ*L of AgPath One-step RT-PCR buffer (Ambion), 0.6 *μ*L of 25x reverse transcriptase, 0.3 *μ*L (45 *μ*M) of each primer, 0.3 *μ*L of 12.5 *μ*M TaqMan probe, and 1 *μ*L of nuclease-free water. The qPCR assays were performed under the following conditions: 10 min at 45°C, 10 min at 95°C, then 45 cycles of 95°C for 15 s, 60°C for 45 s, using a 7500 real-time PCR system (Applied Biosystems).

### 2.6. Virus Culture and Isolation

At BSL, virus isolation was attempted on unpreserved liver and spleen samples (Cases 2–4) using chick embryo fibroblasts (CEF). Culture supernatant from Case 4 was subjected to total nucleic acid extraction using the MagMAX CORE Nucleic Acid Purification Kit (Thermo Fisher Scientific) with a KingFisher Duo Prime Purification System (Thermo Fisher Scientific). Two hundred microlitres of supernatant were added to the lysis buffer mix for robotic extraction as per the manufacturer's instructions. Extracted template was run on previously described CoHV-1 PCR [25] and Goose herpesvirus PCR [26] using standard cycling conditions of 95°C for 5 min followed by 35 cycles of 95°C for 30 s, 55°C for 30 s, and 72°C for 20 s, before a hold at 72°C for 5 min.

At ACDP, two virus isolation methods were used, inoculation into Muscovy Duck Embryo Fibroblast (MDEF) cells, and specific pathogen free (SPF) chicken eggs. MDEF cells were cultured in Eagle's minimum essential medium (EMEM) supplemented with 10% foetal bovine sera (FBS), 2 mM glutamine, 10 mM HEPES, 100 U/mL penicillin, 100 *μ*g/mL streptomycin (all from Thermo Fisher Scientific, Australia) and 0.17% NaHCO_3_ (sodium bicarbonate, Merck, Australia). Cells were cultured at 37°C with 5% CO_2_. For virus isolation, fresh spleen and liver samples were collected from diseased birds and transported on ice to the laboratory. A 10% homogenate was prepared in sterile phosphate-buffered saline (PBS) and spun at high speed in a microfuge to clarify. The MDEF cells were then prepared by culturing in 2% FBS media with all remaining supplements the same as above. Five hundred microlitres of clarified sample were added to confluent MDEF cells in 25 cm^2^ flasks and incubated at 37°C with 5% CO_2_ for 1 hr. After 1 hr adsorption, 9.5 mL of supplemented media with 2% FBS was added. Flasks were incubated 37°C with 5% CO_2_ for 7 days with daily monitoring for CPE.

### 2.7. Electron Microscopy

Formalin-fixed tissue samples (Cases 3 and 4) were initially sectioned to generate two halves, with one destined for routine histology processing and haematoxylin and eosin staining, and the other for transmission electron microscopy (TEM). Once a suitable region was identified on histopathology, the corresponding region on the opposite half of the fixed sample for TEM was excised to approximately 1 mm^3^ cube. The samples were then conventionally processed into Embed 812 resin, sectioned, and stained, as previously described [27].

For the FFPE liver sections (Case 1), standard 4-*µ*m thick sections were taken from histology blocks, mounted on Thermanox coverslips (Product code GL084, ProSciTech Kirwan, QLD, Australia), dewaxed in xylene and rehydrated before processing into Spurr's resin, as previously described [28]. Immediately after polymerisation, while the blocks were still hot, the coverslips were quickly torn off, which left behind the visible section on a smooth surface that was then correlated to the region of interest, trimmed, sectioned, and stained as above.

Virus inoculated MDEF cells were pelleted in the growth media, the supernatant removed and replaced with 2.5% glutaraldehyde in 0.1 M Sorenson's phosphate buffer overnight at 4°C, and then prepared for TEM as described above through to Spurr's Resin [28]. MDEF cells, inoculated with *in vitro* virus, were pelleted in the growth media, the supernatant removed and replaced with 2.5% glutaraldehyde in 0.1 M Sorenson's phosphate buffer overnight at 4°C, and then prepared for TEM, as described above through to Spurr's Resin [28].

Negative contrast TEM was performed on the culture supernatant of inoculated CEF (cultured at BSL; Case 2) and MDEF cells (cultured at ACDP; Cases 3 and 4). The supernatant was adsorbed onto carbon-coated formvar copper grids and then stained with nanoW (Nanoprobes, NY, USA) for 1 min.

All images were acquired using a JEOL JEM-1400 120KV TEM and Gatan Ultrascan 1000 camera.

### 2.8. High Throughput Sequencing

DNA was extracted from the viral cultures using the MagMAX-96 Viral RNA Isolation Kit and processed with the MagMAX Express 24 (ThermoFisher) and sequenced using both short-read Illumina and long-read Oxford Nanopore technologies. For Illumina sequencing, the Nextera XT DNA Library Prep Kit was used to create libraries, which were sequenced on an Illumina MiSeq (150 bp PE reads). For Oxford Nanopore sequencing, the Rapid PCR Barcoding Kit (SQK-RPB004) was used to generate libraries, which were sequenced using an Oxford Nanopore MinION and an R9.4.1 flow cell.

### 2.9. Whole Genome Assembly

The raw Illumina reads were cleaned by removing adapters, trimming sequences with a phred quality score below 20, and discarding any remaining reads shorter than 50 bp with Trimmomatic v.0.39 [29]. Sequences originating from the Muscovy duck cell line were filtered using *bbduk.sh* against the *Anas platyrhynchos* genome (GenBank Accession GCF_015476345.1) with bbmap v.38.96 (sourceforge.net/projects/bbmap/). The raw Oxford Nanopore reads were processed by removing adapters and barcodes with Porechop v.0.2.1 [30]. The cleaned reads were then mapped to the *A. platyrhynchos* genome using MiniMap2 v.2.16 [31] and nonmapping reads were extracted with SAMtools v.1.10.0 [32].

The cleaned and filtered Illumina and Oxford Nanopore reads were combined and assembled using Unicycler v.0.4.8 [33] with default parameters. This resulted in a small number of contigs that were further refined in Geneious Prime v.2020.2.4. Joins between the unique and repeated genome sections were manually examined by iterative mapping of the long and short reads in Geneious Prime and closed where possible.

### 2.10. Gap Closing Conventional PCR and Long-Read Amplicon Sequencing

A small number of connections between genome segments could not be resolved by untargeted sequencing and several conventional PCRs were used to close the genomes (Table 1). For AnHV-3, this included the unique long (U_L_) to internal short repeat (IR_S_) connection and a section of the IR_S_ and terminal short repeat (TR_S_) fragments. The U_L_ to IR_S_ sequence was targeted by the primers swan_UL_F2 (5′-GAT GGT GTA GAG CGA CCG AG-′3) and swan_UL_R2 (5′-CAC GCC TTC TTC ATT ACG CG-′3). The IR_S_ and TR_S_ gaps were targeted by the primers swan_IRS_F1 (5′-TGG GAG GGG GAG TGA ATA GG-′3) and swan_IRS_R1 (5′-CGG AGG AGC ATC TAA GGC TG-′3). For AnHV-2, the U_L_ to IR_S_ join was confirmed by the primers GHV_UL_F2 (5′-GCA TTC CGC ATC CGA AAG TC-′3) and GHV_UL_R2 (5′-GGA CGC GTA TGA GGG AAG AG-′3).

The PCR reactions were composed of 12.5 *µ*L of 2x HotStarTaq (Qiagen), 1 *µ*L of each forward and reverse primer, 5 *µ*L of virus DNA, and 5.5 *µ*L of nuclease-free water to a final volume of 25 *µ*L. The products were amplified with 1 cycle of 94°C for 15 min; 35 cycles of 94°C for 60 s, 56°C for 2 min, 72°C for 90s; then 72°C for 10 min. Amplified products were gel extracted, purified using the QIAquick Purification kit (Qiagen), and quantified with the Qubit dsDNA HS Assay Kit (ThermoFisher). Due to the length of the amplicons (up to 3.5 kb), they were sequenced as complete molecules using Oxford Nanopore long-read sequencing. First, adapters and barcodes were added to the amplicons using the Ligation Sequencing Kit (SQK-LSK109) following the recommendations for amplicons. The prepared fragments were then sequenced using the Oxford Nanopore MinION with an R9.4.1 flow cell. The raw reads were cleaned with Porechop v.0.2.1 [30], and the amplicon data were manually used to close gaps in the genomes with Geneious Prime v.2020.2.4. The completed AnHV-2 and AnHV-3 genomes were then annotated using DRAM v.1.2.1 [34] using default parameters.

### 2.11. Phylogenetic Analysis

A phylogenetic reconstruction was completed according to ICTV guidelines (ICTV, 2021). Several conserved proteins were extracted from the genomes, including uracil-DNA glycosylase (UL2), helicase-primase helicase subunit (UL5), DNA packaging terminase subunit 1 (UL15), major capsid protein (UL19), envelope glycoprotein B (UL27), and DNA polymerase catalytic subunit (UL30). The amino acids from these proteins were concatenated together and combined with proteins from all currently described alphaherpesvirus species (ICTV, 2021). The sequences were then aligned using MAFFT v.7.490 [35] with the *auto* option to choose the most appropriate alignment parameters. IQ-TREE v.2.2.0.5 [36] was used to create a midpoint rooted, maximum likelihood phylogenetic tree with the “Q.insect+F+I+G4” model chosen as the most appropriate. A phylogenetic reconstruction specifically comparing AnHV-2 strains was also conducted as above, except that nucleotides from the genes were used and the “TVM+F+G4” evolutionary model was selected. Both trees were annotated and rendered using the R package ggtree v.1.14.6 [37].

## 3. Results

### 3.1. Case Presentations

Historical case: In January 1989, in south-eastern Queensland, an outbreak of disease resulted in the death of 76 geese from a flock of 78 over a 24-day period. A herpesvirus was isolated during the investigation [15] and further work on the isolate (GHV 552/89) was conducted by Gough and Hansen [16], although genomic sequence was not obtained.

Case 1: In February 2013 in Townsville, Queensland, a property experienced morbidity of two of five domestic geese and one of four domestic chickens housed outdoors. Both affected geese were aged approximately 2 years old. Geese and chickens had access to four small ponds of fresh bore water, also accessed by wild ducks. The affected geese died within 24 hr of onset of clinical signs. One goose received treatment at the local emergency hospital and died while hospitalised.

Case 2: In January 2017 on a property in Maryborough, Queensland, 13 of 19 mixed-age geese died over a 10-day period. Geese were housed outdoors with ducks, turkeys, and chickens unaffected by illness, and the domestic population co-mingled with wild ducks.

Case 3: In February 2020 in Townsville, Queensland, a property reported the deaths of two of three domestic geese. The property had previously experienced sporadic deaths of one to two geese every summer since 2016. Geese deaths re-occurred annually in the wet season of November to February, during or after periods of extreme heat and humidity followed by tropical monsoon rains and the arrival of hundreds of migratory waterfowl to the property. Affected geese died within 24 hr from the onset of clinical signs. Geese were housed outdoors and co-mingled with both hand-reared domestic and wild ducks, magpie geese and flocks of hundreds of wild migratory ducks, magpie geese, and straw-necked ibis which were unaffected by the illness.

Case 4: In February 2021 in Lockyer Valley, Queensland, 48 of a flock of 50 geese on a hobby farm died over a period of 4 weeks. All geese were adults over 1 year of age and were housed outdoors with domestic ducks, chickens, and turkeys which were unaffected by disease. The flock had contact with wild ducks and magpie geese. Affected geese died within 24 hr from the onset of clinical signs and were often found dead next to or in water troughs.

Swan case: An archived case from February 1991 of a black swan with similar presentation and pathology to the original goose herpesvirus outbreak in 1989 was extracted from the ACDP database. The black swan was located on a rainforest reserve in Cairns, Queensland, and was found dead. Post-mortem findings suggested herpesvirus infection, and an unknown herpesvirus was cultured at the time.

### 3.2. Clinical Signs and Pathology

In all outbreaks, affected geese displayed similar clinical signs, including anorexia, vomiting, regurgitation, diarrhoea, mydriasis, conjunctivitis, lethargy, weakness progressing to ataxia, prostration, and recumbency (Table S1).

**Figure 1:**
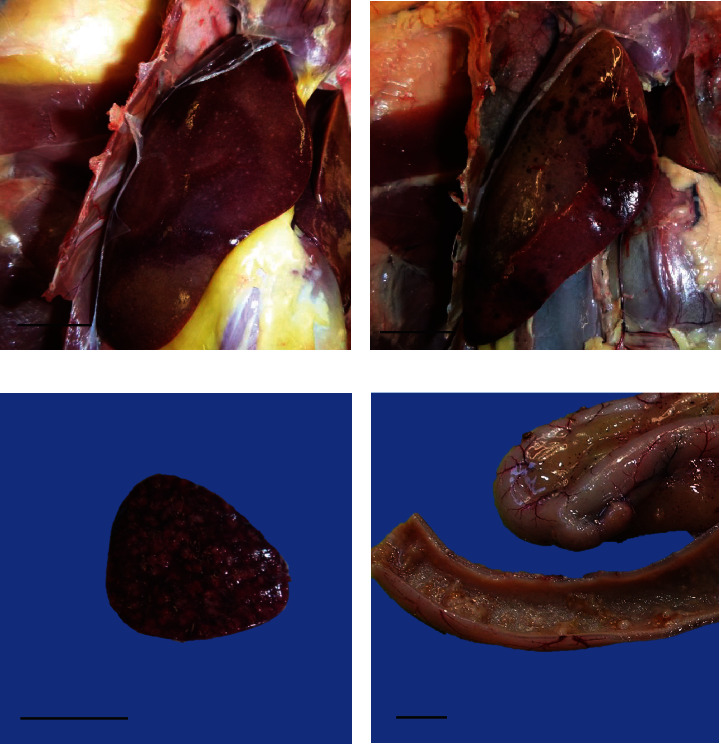
Necropsy photos of tissues from Cases 3 and 4. (a) Liver from a goose from Case 4 with multifocal pinpoint tan lesions. Scale bar: 2 cm. (b) Liver from a goose from Case 4 with multifocal to coalescing haemorrhagic necrosis. Scale bar: 2 cm. (c) Spleen from a goose from Case 4 showing multifocal to coalescing pale areas of necrosis. Scale bar: 1 cm. (d) Small intestine from Case 3 displaying plaques and fibrinonecrotic material on the mucosal surface, congestion of the serosal vessels, and petechial haemorrhage of the mesenteric adipose tissue. Scale bar: 1 cm.

Similar lesions of varying severities were observed in the geese that underwent necropsy (Figure 1). The liver contained generalised, pinpoint tan foci, or multifocal dark red haemorrhages (Figures 1(a) and 1(b)). The spleen was frequently enlarged and exhibited multifocal pinpoint to 2 mm tan areas on the cut surface (Figure 1(c)). In the small intestine, there were multifocal to coalescing, grey to tan, raised plaques on the mucosal surface (Figure 1(d)). A few geese also displayed petechial haemorrhages on serosal surfaces and in coelomic adipose tissue (Figure 1(d)). See Table S1 for pathologic features observed in each case.

**Figure 2:**
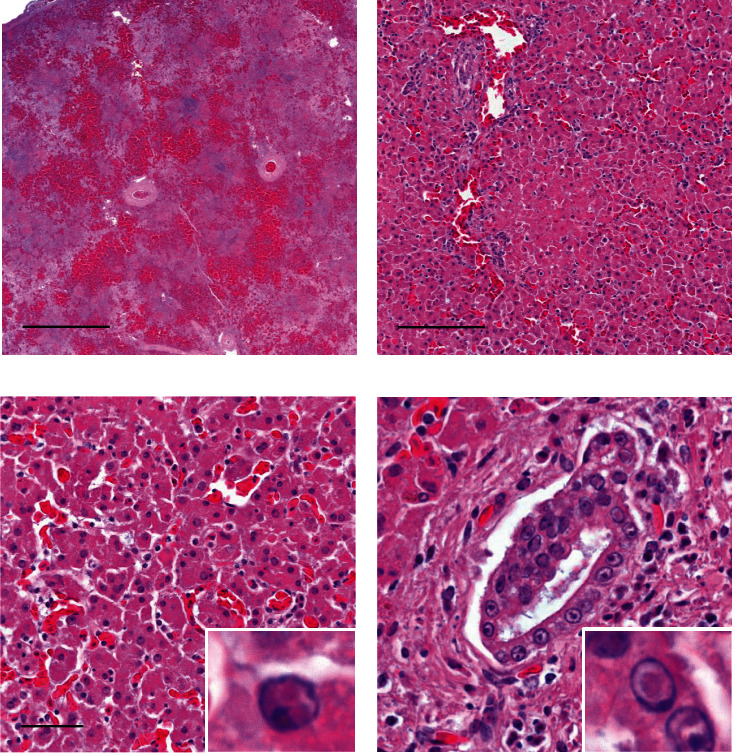
Photomicrographs of tissues from Case 4, stained with haematoxylin and eosin. (a) Low power view of spleen showing multifocal to coalescing necrosis and lack of lymphoid tissue. Scale bar: 1 mm. (b) Section of liver with multifocal, randomly distributed necrosis. Scale bar: 100 *µ*m. (c) Higher power view of the liver showing an area of necrosis, characterised by loss of cellular detail, karyorrhexis, cytoplasmic eosinophilia, and infiltration by heterophils. Hepatocytes frequently contain eosinophilic intranuclear inclusion bodies, as shown in higher magnification in the inset. Scale bar: 50 *µ*m. (d) Section of liver with a bile duct, which contain intranuclear inclusion bodies in the biliary epithelial cells. Scale bar: 30 *µ*m.

Histopathology of the livers showed multifocal, randomly distributed necrosis, accompanied by haemorrhage in the more severely affected cases (Figures 2(b) and 2(c)). Hepatocytes surrounding areas of necrosis frequently contained single or multiple, round, 2–6 *μ*m, amphophilic to eosinophilic intranuclear viral inclusions surrounded by a clear halo which often marginated the chromatin (Figure 2(c)). Inclusions were also present in biliary epithelium (Figure 2(d)). The small intestine exhibited fibrino-necrotising enteritis, with multifocal to coalescing necrosis of villi and crypts, and intranuclear inclusions in intact mucosal epithelial cells. Some geese also displayed necrosis of small intestinal lymphoid tissue. There was necrotising splenitis with necrosis and depletion of lymphoid tissue (Figure 2(a)). Other changes which occurred in a few birds included necrotising thymitis, necrotising adrenalitis, vasculitis, and further intranuclear inclusion bodies were detected in the thymus, mucous gland epithelial cells of the crop, respiratory epithelium and vascular endothelium of the lungs, ovarian stromal cells, adrenal cortical cells, and neuronal cell bodies in peripheral nerve ganglia.

### 3.3. Molecular Diagnostics

Based on the presentation and clinicopathologic findings, a viral causative agent was highly suspected. Real-time PCR was performed on one or a combination of cloacal swab, tracheal swab, fresh liver, and fresh spleen from each case other than Case 1. Samples were negative for Newcastle disease virus classes I and II, Avian influenza type A, duck enteritis virus (AnHV-1), duck hepatitis virus 1, and pigeon rotavirus. Pan-herpesvirus PCR was positive on liver and spleen samples from Case 4. Analysis of this short sequence revealed the presence of a novel herpesvirus, which was further investigated using viral isolation, microscopy, and whole genome sequencing. Following sequencing and development of an AnHV-2-specific qPCR assay, Cases 1, 3, and 4 tested positive. See Table S2 for an overview of the diagnostic tests performed on each case.

### 3.4. Virus Isolation and Transmission Electron Microscopy

Virus isolation was attempted at BSL and ACDP using CEF and MDEF cells, respectively. At BSL, an unidentified virus was cultured with cytopathic effect (CPE) observed in liver samples from two birds of Case 2. From Case 3, an unidentified virus was cultured with CPE observed from liver and spleen samples. From Case 4, no virus was isolated from liver but an unidentified virus was cultured from spleen resulting in CPE. At ACDP, MDEF cells inoculated with 10% homogenates of liver and spleen samples from Cases 3 and 4. CPE was observed in inoculated cells 24 hr post-inoculation for both Cases 3 and 4. MDEF cells were infected with a 1 : 10,000 dilution of supernatant to obtain a culture suitable for analysis by TEM analysis.

**Figure 3:**
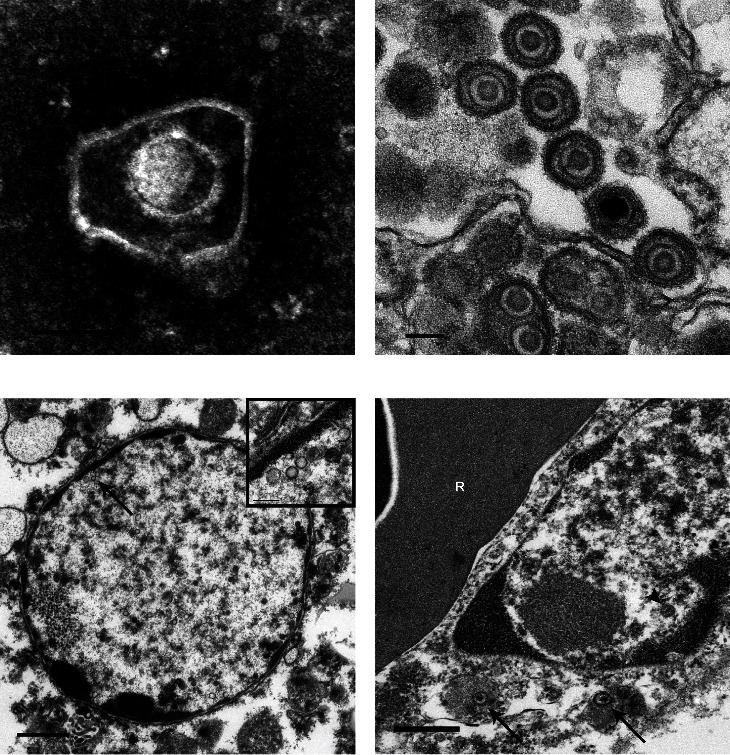
Transmission electron microscopy of inoculated MDEF cells and tissues from Cases 3 and 4. (a) Case 4, negative contrast electron microscopy of culture supernatant from inoculated MDEF cells showing enveloped viral particle containing an icosahedral capsid, morphology consistent with virus belonging in the family *Herpesviridae*. Scale bar: 100 nm. (b) Case 4, thin section electron microscopy of inoculated MDEF cells showing multiple herpesviral particles in the cytoplasm of a degenerating cell. Scale bar: 100 nm. (c) Case 3, thin section electron microscopy of a hepatocyte from a Case 3 goose showing a nucleus containing many herpesviral capsids with and without an electron-dense core (higher magnification of area indicated by the arrow is shown in the inset). Scale bar: 1 *µ*m for panel (c); 200 nm for panel (c) inset. (d) Case 4, thin section electron microscopy of an endothelial cell in the liver of a Case 4 goose showing herpesviral particles in the cytoplasm (arrows) and nucleus (arrowhead). “R” denotes nucleated red blood cell located in the lumen of the blood vessel, immediately adjacent to the endothelial cell. Scale bar: 500 nm.

Infected cultures from Cases 2–4, virus isolations contained viral particles with morphology consistent with those of the family *Herpesviridae*. On negative contrast TEM, the viral particles were enveloped and contained an icosahedral nucleocapsid (Figure 3(a)). On thin section TEM, herpesviral capsids were identified within the nucleus, often scattered in the periphery of the nucleoplasm subjacent to the nuclear membrane, and in the cytoplasm (Figure 3(b)), often within rough endoplasmic reticulum and occasionally clustered within masses of non-membrane bound homogeneous electron-dense material in the cytosolic matrix, reminiscent of “dense bodies” reported in cytomegalovirus infected cells [38].

TEM was also performed on formalin-fixed tissue samples from geese of Case 4 (liver only) and Case 3 (liver, lung, spleen, and small intestine), as well as formalin-fixed paraffin-embedded (FFPE) liver sections from Case 1. Herpesviral particles were observed in all liver samples submitted. Viral capsids with or without electron-dense cores were often seen in the nuclei of hepatocytes (Figure 3(c)), and to a lesser extent, biliary epithelial, and endothelial cells (Figure 3(d)). Similarly, viral particles were identified in endothelial cells of a large splenic artery. Viral capsids were also seen in nuclei of several unidentifiable cells in the liver, spleen, and small intestine. These cells could not be identified with confidence due to poor cellular and tissue architectural preservation.

### 3.5. Genome Sequencing

Whole genome sequencing was attempted on viral cultures obtained from the historical case, the swan case, Cases 2–4, and from formalin-fixed tissue from Case 1. Complete genomes were obtained from each case, except for Case 1 (Townsville 2013), where suitable sequence could not be obtained from the fixed tissues.

**Figure 4:**
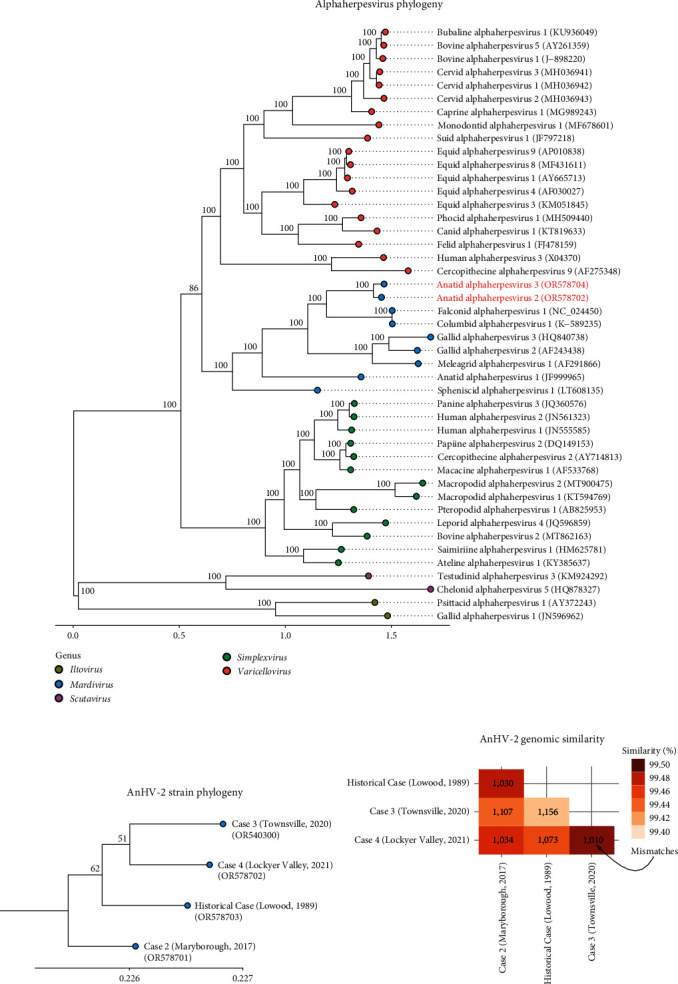
Maximum likelihood phylogenetic tree of conserved alphaherpesvirus proteins (a), maximum likelihood phylogenetic tree comparing the four AnHV-2 genomes (b), and genomic similarity between the four AnHV-2 genomes (c). The results from 1,000 bootstrap replicates are given on the nodes (a, b) and the scale represents the number of amino acid (a) or nucleotide (b) substitutions per site.

**Figure 5:**
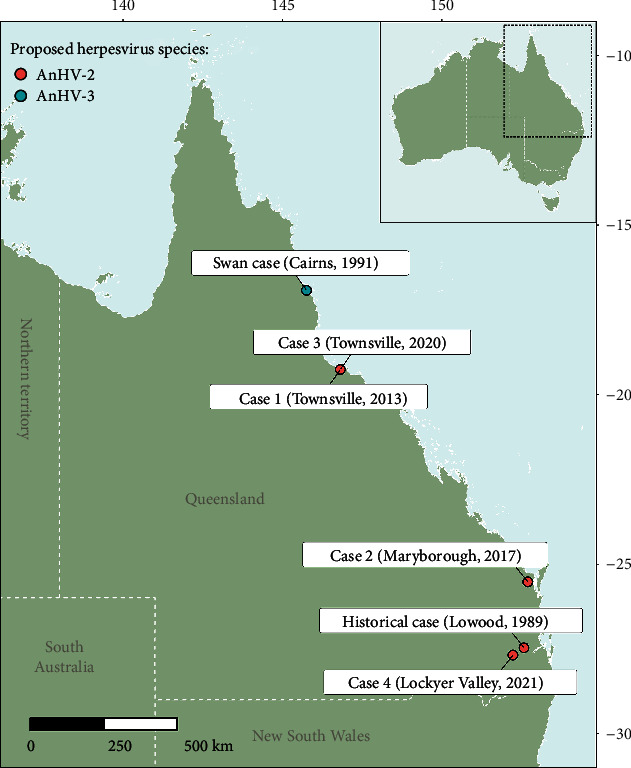
Anatid alphaherpesvirus 2 and 3 case locations, collection year, and viral species detected. Decimal coordinates are given on the *x*- and *y*-axes.

Genome sequencing demonstrated the presence of two distinct herpesvirus species (Figure 4(a)), preliminarily designated anatid alphaherpesvirus 2 (AnHV-2) and anatid alphaherpesvirus 3 (AnHV-3). All AnHV-2 detections were from geese samples, while AnHV-3 was detected in a single swan from its only known occurrence in Cairns 1991 (Figure 5). Although these two alphaherpesvirus genomes were more similar to each other than to reference sequences, they had a nucleotide identity of only 76.1%, necessitating their delineation.

**Figure 6:**
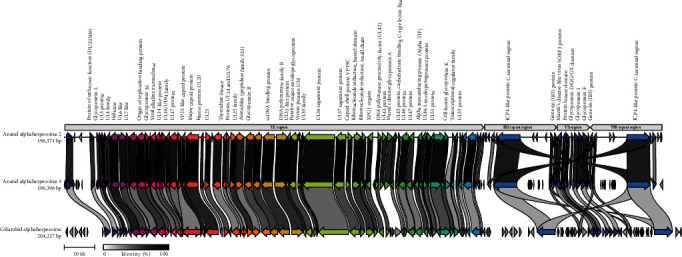
Gene location and structural genome arrangement of anatid alphaherpesvirus 2 (OR578702), anatid alphaherpesvirus 3 (OR578704), and Columbid alphaherpesvirus 1 (NC_034266). Genes with the same colour indicate homologues and grey bars connecting the genes indicate the level of similarity.

The AnHV-2 genome was approximately 196,000 bp with a GC content of 62.2% and 100 predicted open reading frames (ORFs), while AnHV-3 had a 196,396 bp genome with a GC content of 57.8% and 123 ORFs (Figure 6). The discrepancy in the number of ORFs was due to a larger number of hypothetical proteins in AnHV-3. Nevertheless, both species had similar genomic arrangements and contained all conserved herpesvirus genes (Figure 6). In terms of overarching genome structure, AnHV-2 and AnHV-3 formed the typical class 3 herpesvirus genome arrangement of TR_L_-U_L_-IR_L_-IR_s_-U_S_-TR_S_ (Figure 6). Both genomes contained a putative origin of replication (OriS) comprised of an almost perfect 61 bp palindrome that only differed by 3 bp in AnHV-2 and AnHV-3.

### 3.6. Phylogenetic Analysis

To clarify the phylogenetic position of AnHV-2 and AnHV-3 in relation to other alphaherpesviruses, several conserved protein sequences were compared to reference sequences (Figure 4(a)). The new genomes formed a distinct clade within the alphaherpesvirus genus *Mardivirus* and were most similar to other avian viruses from pigeons, falcons, chickens, turkeys, and penguins (Figure 4(a)). Although similar to each other, the branch distances of AnHV-2 and AnHV-3 confirmed their classification as distinct species.

Four genomes of AnHV-2 were assembled, spanning a period of more than 30 years. Phylogenetically, the 4 AnHV-2 genomes did not exhibit a clear temporal signal. For example, the oldest genome from Lowood in 1989 was not more divergent when compared to the contemporary genomes (Figure 4(b)). In fact, each of the AnHV-2 genomes contained a relatively consistent number of variations of between 1,010 and 1,156 positions when compared to each other (Figure 4(c)). The AnHV-2 genomes also did not display any apparent geographic clustering within samples collected from southern or northern Queensland (Figures 4(b) and 4(c)).

## 4. Discussion

This paper describes several outbreaks of naturally occurring goose herpesvirus (AnHV-2) causing morbidity and mortality in domestic geese over several decades in Queensland, Australia. The pathologic findings of necrotising hepatitis and splenitis, and the identification of herpesvirus particles in hepatocytes, biliary epithelium, and endothelial cells by TEM are consistent with what has been described for alphaherpesviral infection in other species [13, 39, 40]. Using whole genome sequencing, and a subsequently developed specific RT-PCR for AnHV-2, the historical outbreak of “goose herpesvirus” in 1989 (e.g., [15]) was linked to contemporary outbreaks in 2013, 2017, 2020, and 2021. The virus likely responsible for these outbreaks was herein designated anatid alphaherpesvirus 2 (AnHV-2), consistent with the nomenclature of herpesviruses that infect anatid species. During the investigation, another herpesvirus isolated from a swan in 1991 was also sequenced. Genome sequencing revealed this was a second distinct species, preliminarily designated as anatid alphaherpesvirus 3 (AnHV-3).

The two herpesviruses were most closely related to Columbid alphaherpesvirus 1 (CoHV-1), which is also known as pigeon herpesvirus (PiHV) and has been detected in multiple avian species, including raptors [10, 11, 12]. Despite being the most similar reference available, CoHV-1 only had a nucleotide identity of approximately 47.5% with AnHV-2 and AnHV-3, indicating a distant evolutionary history. Anatid herpesvirus 1 (AnHV-1), the cause of duck plague, was previously found to be genetically distinct from AnHV-2 (then termed GHV 552/89) using restriction enzyme patterns [16]. This was confirmed here using whole genome sequencing, which showed a nucleotide similarity of less than 46% between AnHV-1 and the newly designated AnHV-2 and AnHV-3.

The more recent outbreaks of AnHV-2 occurred sporadically in the months of January and February over a period of 8 years. The historic outbreak reported by Ketterer et al. [15] occurred 24 years prior in 1989, also in January. All described outbreaks to date have been distributed along the eastern seaboard of Queensland, which incorporates subtropical and tropical climates, and spans over 1,600 km. The spatiotemporal distribution could suggest overlapping of multiple factors are required to result in disease, such as changes in rainfall or ambient temperatures, increased population density, increased co-mingling of wild and domesticated birds at water sources, and breeding season for geese. In all the outbreaks, geese had a history of contact with wild Anseriformes and other birds, which could be an original source of the herpesvirus. Surveillance of domestic and wild birds in the region of the previously reported outbreak in 1989 did not identify goose herpesvirus infection in these populations [15], although testing was not exhaustive. It is suspected that an as yet unidentified wild bird, possibly from the *Anatidae* family, is the reservoir host of AnHV-2, and that this host interacts with domestic geese over the Australian summer months, facilitating spill-over of the virus.

Indeed, recent surveillance of wild bird populations in Australia and overseas has demonstrated several novel herpesviruses, some of which were detected across more than one avian order [3, 4, 5]. For example, Amery-Gale et al. [4] detected two novel Australian alphaherpesviruses in a sulphur-crested cockatoo and a tawny frogmouth, tentatively named Cacatuid alphaherpesvirus 1 and Podargid alphaherpesvirus 1. Only a small portion of the DNA-dependant DNA polymerase gene is available for these viruses, which ranges from 53.3% to 64.6% nucleotide identity to AnHV-2 and AnHV-3. Thus, although these viruses are unlikely to be directly related, it does imply a largely undiscovered virodiversity in wild Australian birds, and a precedent for crossing the species barrier which could impact domestic species or endangered wildlife.

Latent or chronic infections in a domestic or sylvatic transmission cycle could also play a role in AnHV-2 outbreaks. In DVE, AnHV-1 becomes latent in the trigeminal ganglion and lymphoid tissues, and re-activation of the virus has been demonstrated to cause outbreaks in domestic and migrating Anseriformes [13]. A similar process may occur in AnHV-2.

The clinical presentations, pathology, and ultrastructural findings were all consistent with the previous report of goose herpesvirus [15]. The current study identified a wider range of lesions associated with infection. In addition to the previously reported necrotising hepatitis and enteritis, there was necrotising splenitis, thymitis, adrenalitis and vasculitis, and intranuclear inclusion bodies were observed in a wider range of organs. Comparable clinicopathologic findings are reported in other acute systemic herpesviral infections in birds, such as DVE, Pacheco's disease (psittacine alphaherpesvirus 1), and pigeon herpesvirus [10, 12, 13, 40, 41]. Similar lesions are also found in systemic herpesviral infections of mammals, for example, canine herpesvirus infection in neonatal puppies [39]. It is feasible AnHV-2 shares a similar pathogenesis with these herpesviruses that cause systemic infection.

This report of AnHV-2 has implications for goose flock health and production output, particularly with the rise of domestic goose farming. Extensive surveillance and outbreak testing for AnHV-2 should be performed in other Australian states and worldwide, to determine the distribution of infection. It is important for owners/producers and veterinary professionals to be aware of this herpesvirus. In Australia, DVE (AnHV-1) is considered exotic and is notifiable. Thus, differentiating AnHV-1 and AnHV-2 in outbreaks of Anseriforme mortality in Australia is of critical importance.

## 5. Conclusion

High mortality disease events in domestic geese have been reported periodically since 1989 in south-eastern Queensland, Australia. Here, we demonstrate that the same herpesvirus, designated AnHV-2, was the likely cause of these outbreaks in 1989, 2013, 2017, 2020, and 2021. The increasing frequency of these disease outbreaks in domestic geese and the geographical expansion of disease outbreaks to tropical areas of northern Queensland may be a result of shifting climatic conditions leading to changes in bird movement patterns or may simply be the result of higher levels of reporting to government departments. It was hypothesised that black swans (*Cygnus atratus*) were a reservoir host of AnHV-2 due to the detection of a herpesvirus in a swan sample from 1991. However, genomic sequencing revealed that this was another distinct species (named here AnHV-3). Although a reservoir host was not detected here, the case histories suggest a reservoir host that interacts with domestic geese during the Australian summer months, allowing viral spill-over to occur. Expanded surveillance testing of unaffected and affected domestic geese and in contact Anseriformes and wild birds is warranted, to determine the extent of infection and potential reservoir hosts. We developed a specific RT-PCR for the rapid detection of AnHV-2 and generated whole genome resources that will facilitate surveillance and outbreak investigations in the future.

## Figures and Tables

**Table 1:** Primer sequences for the AnHV-2 RT-PCR and the conventional PCRs used to close gaps in the genome assemblies.

Name	Target	Sequence
GHV-F	AnHV-2 RT-PCR	5′-GGC GAA CGG CCT GTT G-3′
GHV-R	AnHV-2 RT-PCR	5′-TGA ATA TAG TCG CGT ACC GAC AA-3′
GHV-Probe	AnHV-2 RT-PCR	5′- FAM—ACC GTC ACC/ZEN/ACG ATA GGT CGC AAC A-3′-IABkFQ-5′
GHV_UL_F2	AnHV-2 U_L_-IR_s_	5′-GCA TTC CGC ATC CGA AAG TC-′3
GHV_UL_R2	AnHV-2 U_L_-IR_s_	5′-GGA CGC GTA TGA GGG AAG AG-′3
swan_UL_F2	AnHV-3 U_L_-IR_s_	5′-GAT GGT GTA GAG CGA CCG AG-′3
swan_UL_R2	AnHV-3 U_L_-IR_s_	5′-CAC GCC TTC TTC ATT ACG CG-′3
swan_IRS_F1	AnHV-3 IR_s_ TR_s_ gaps	5′-TGG GAG GGG GAG TGA ATA GG-′3
swan_IRS_R1	AnHV-3 IR_s_ TR_s_ gaps	5′-CGG AGG AGC ATC TAA GGC TG-′3

## Data Availability

The complete genome sequences were deposited in NCBI's GenBank under accession numbers: OR578701 (Case 2), OR540300 (Case 3), OR578702 (Case 4), OR578703 (historical case), and OR578704 (swan case).

## Supplementary Materials

Supplementary Materials Supplementary materials provide detailed information regarding the epidemiologic and clinicopathologic features and diagnostic tests performed for each case.
