# Supplementary material for: Multidecadal High Mortality Disease Events in Australian Domestic Geese Associated with a Novel Alphaherpesvirus, Designated Anatid Alphaherpevirus 2
Source: Transbound Emerg Dis. 2024 Jun 7;2024:3255966. doi: 10.1155/2024/3255966 (PMC12020334; doi:10.1155/2024/3255966)
Supplement: Supplementary Materials — provide detailed information regarding the epidemiologic and clinicopathologic features and diagnostic tests performed for each case. [file 3255966.f1.docx]

Supplementary Table 1: Summary of epidemiologic and clinicopathologic findings for each case.

|  | **Epidemiology** | | | | | | **Clinicopathologic findings** | | |
| --- | --- | --- | --- | --- | --- | --- | --- | --- | --- |
|  | **Date** | **Location** | **Species affected** | **Number at risk** | **Number dead** | **Age** | **Clinical signs** | **Gross pathology** | **Histopathology** |
| Historical case [15] | 15/01/1989 | Lowood | Domestic goose (*Anser anser domestics*) | 78 | 76 | 1-2 years | Weakness, prostration, and rapid death. | Reasonable body condition. Livers varied from pale and small to enlarged and congested with scattered multifocal petechial haemorrhage and numerous pinpoint white foci on the capsular surface. The small intestine contained white floccular fibrinous exudate, and the mucosa was hyperaemic with scattered 1 mm diameter, raised nodular brown-white foci and several raised plaques of 5 mm diameter. | Multifocal hepatic necrosis with hepatocellular eosinophilic intranuclear inclusion bodies, and fibrinous enteritis with villi and crypt necrosis and lymphoid necrosis |
| Case 1 | 9/02/2013 | Townsville | Domestic goose (*Anser anser domestics*) | 5 | 2 | 2 years | Lethargy, recumbency, vomiting, open-mouth breathing, stiff gait, dehydration, and death within 24 hours. | Good body condition. The crop contains green ingesta. Petechiae and ecchymotic haemorrhages noted in proventriculus. The liver was pale with multifocal petechiae, and petechiae were also present on the serosal surface of the small and large intestine (< 10%). The spleen was mottled. | Multifocal to coalescing necrosis of the spleen, thymus, and intestinal lymphoid tissue, and mild to moderate multifocal hepatic necrosis. Moderate to marked, multifocal erosion and epithelial ballooning degeneration with mucous gland necrosis of the crop. Eosinophilic intranuclear inclusion bodies observed in hepatocytes, spleen, heart, thymus, adrenal gland, lung and ganglion. |
| Case 2 | 10/01/2017 | Maryborough | Domestic goose (*Anser anser domestics*) | 19 | 13 | Adult | Lethargy, recumbency, open-mouth breathing, diarrhoea, tremors, and death within 12 hours. | Unknown | Multifocal splenic and hepatic necrosis |
| Case 3 | 6/02/2020 | Townsville | Domestic goose (*Anser anser domestics*) | 3 | 2 | 5 months | Lethargy, inappetence, vomiting, diarrhoea, and death within 24 to 48 hours. | Moderate body condition, with faecal staining around the cloaca and conjunctivitis in the left eye. The oesophagus and proventriculus were markedly distended with watery green fluid contents. The small intestine contained watery green brown contents, and there were multifocal, raised, grey, 1 to 2 mm plaques on the mucosal surface of small intestine. There were multifocal petechial haemorrhages on the capsular surface of the liver, in the mesenteric adipose tissue, and on the epicardium. There was thymic enlargement, and splenomegaly with a marbled appearance. | Multifocal to coalescing necrotising enteritis, multifocal necrotising hepatitis with vasculitis, multifocal necrotising splenitis with lymphoid depletion, diffuse pulmonary congestion, necrotising thymitis with vasculitis, and adrenalitis. Eosinophilic intranuclear inclusion bodies observed in the hepatocytes, spleen, thymus, adrenal gland, respiratory epithelium and endothelial cells in lung, mucosal epithelium of the oesophagus, crop, proventriculus, intestine, stromal cells of the ovary and ganglia near the cloaca. |
| Case 4 | 5/01/2021 | Lockyer Valley | Domestic goose (*Anser anser domestics*) | 50 | 48 | 1-2 years | Lethargy, inappetence, ataxia, and death within 24 hours. | Good body condition. The liver contained generalised pinpoint tan foci to multifocal dark red (haemorrhagic) lesions. There was splenomegaly with multifocal pinpoint to 2 mm tan areas on cut surface, and the small intestine contained multifocal to coalescing grey-tan raised plaques or nodules on mucosal surface. | Acute multifocal to coalescing, random, necrotising to necrohaemorrhagic hepatitis with hepatocellular and cholangiocellular intranuclear inclusions, acute multifocal random necrotising splenitis with lymphoid necrosis and depletion, acute focal necrotising enteritis with epithelial intranuclear inclusions. |
| Swan case | 14/02/1991 | Cairns | Black swan (*Cygnus atratus*) | 4 | 2 | Adult | Found dead with no observed preceding clinical signs. | Unknown | Splenic and hepatic necrosis with intranuclear inclusions seen in liver |

Supplementary Table 2: Summary of diagnostic tests performed for each case.

|  | **Molecular tests/serology** | **Virus isolation** | **AnHV-2 specific PCR** | **Electron microscopy** | **Whole genome sequencing** | **Other tests** |
| --- | --- | --- | --- | --- | --- | --- |
| Historical case [15] | Serum neutralisation negative against several DVE isolates, infectious laryngotracheitis (Gallid alphaherpesvirus 1), Pacheco's parrot herpesvirus (Psittacid alphaherpesvirus 1) or pigeon herpesvirus (CoHV-1). | Herpesvirus isolated from liver and intestine | N/A | Icosahedral viral nucleocapsid consistent with herpesvirus were seen in negatively stained liver and cell culture material. In ultrathin sections of liver, aggregates of viral nucleocapsids were observed in the nucleus and enveloped particles were identified in the cytoplasm of hepatocytes. | NGS performed on historic virus isolate sequenced goose herpesvirus (AnHV-2). GenBank #OR578703. | Negative for Salmonella culture, botulism by mouse inoculation, liver lead & arsenic, |
| Case 1 | N/A | N/A | Positive on formalin fixed, paraffin embedded (FFPE) liver | Herpesvirus particles observed in hepatocytes from FFPE liver | NGS was attempted on FFPE liver but suitable sequence was not obtained. | N/A |
| Case 2 | Negative Avian Influenza Type A and Newcastle disease virus (NDV) on PCR (tracheal, cloacal, intestine swabs and fresh liver); negative DVE and pigeon rotavirus PCR (fresh liver) | Herpesvirus isolated from fresh liver | N/A | Herpesvirus particles observed on negative contrast electron microscopy of tissue culture supernatant. | NGS performed on tissue culture supernatant obtained sequence >99.5% nucleotide similarity to Historical case (AnHV-2). GenBank #OR578701. | Negative aerobic and Salmonella sp. culture (fresh kidney and spleen), negative Botulism type C/D ELISA results (crop contents and proventriculus) |
| Case 3 | Negative for DEV, duck hepatitis virus (DHV1), Duck Astrovirus 1 and Duck Astrovirus 2 on PCR (fresh liver and spleen and viral isolates from liver and spleen) | Herpesvirus isolated from fresh liver and spleen | Positive on fresh liver and spleen and viral isolates from liver and spleen | Herpesvirus particles observed on negative contrast (tissue culture supernatant) and thin section electron microscopy of cell culture, and formalin fixed liver, lung, spleen, and small intestine. | NGS performed on tissue culture supernatant obtained sequence >99.5% nucleotide similarity to Historical case (AnHV-2). GenBank #OR540300. | Microbiology cultured *Escherichia coli* and *Streptococcus* sp. (tracheal swab), *Streptomyces* sp. (liver), and *E. coli* and *Clostridium* sp. (duodenum) |
| Case 4 | Negative for NDV class I/II and Avian Influenza type A by PCR (tracheal and cloacal swabs); negative pigeon herpesvirus PCR (viral isolate from spleen); negative DVE and DHV1 PCR (fresh liver and spleen). Positive for Pan herpesvirus PCR (fresh liver and spleen). | Herpesvirus isolated from fresh liver and spleen | Samples were used to develop the AnHV-2 PCR | Herpesvirus particles observed on negative contrast (tissue culture supernatant) and thin section electron microscopy of cell culture and formalin fixed liver. | NGS performed on tissue culture supernatant obtained sequence >99.5% nucleotide similarity to Historical case (AnHV-2). GenBank #OR578702. | N/A |
| Swan case | N/A | Herpesvirus isolated from fresh liver and kidney | N/A | N/A | NGS performed on cell culture identified a distinct herpesvirus with genome identity of 76.1% to AnHV-2 (AnHV-3). GenBank #OR578704. | N/A |
